# Improved recovery of nontuberculous mycobacteria in culture with adjunctive use of a selective agar

**DOI:** 10.1128/jcm.01678-23

**Published:** 2024-02-23

**Authors:** Mikayla Caldwell, Jena Tisdale, Reeti Khare

**Affiliations:** 1 Advanced Diagnostic Laboratories, National Jewish Health, Denver, Colorado, USA; The University of North Carolina at Chapel Hill School of Medicine, Chapel Hill, North Carolina, USA

**Keywords:** nontuberculous mycobacteria, NTM Elite, cystic fibrosis, acid-fast bacilli, culture

## LETTER

NTM Elite (bioMèrieux, France) agar is a selective medium for the isolation of nontuberculous mycobacteria (NTM) from respiratory specimens. Decontamination methods are used to reduce bacterial growth in conventional culture for acid-fast bacilli (AFB), but these techniques can also kill 20%–90% of mycobacteria (1, 2). Conversely, there is no decontamination step prior to inoculating NTM Elite, and the total incubation time is only 4 weeks instead of 6 weeks (3). Here, mycobacterial recovery on NTM Elite was compared to recovery by conventional culture methods.

Prospective respiratory specimens (*N* = 376) from cystic fibrosis (CF, *n* = 156) and non-CF (*n* = 220) patients were plated onto NTM Elite using a swab and incubated in non-CO₂ conditions at 30°C ± 2°C. Plates were read at 4, 7, 14, 21, and 28 days as per manufacturer’s instructions (3). NTM showed a typical appearance on NTM Elite (e.g., rough/smooth and white/yellow), while breakthrough bacterial contaminants appeared blue/red.

All samples were also digested, decontaminated, and inoculated onto Middlebrook-7H11/Mitchison-7H11S agar, Lowenstein Jensen media (LJ, Remel, USA), and Mycobacterial Growth Indicator Tube (MGIT) broth (BD Diagnostics, USA). Solid media were incubated in CO₂ at 37°C ± 2°C and monitored for growth at 1, 3, and 6 weeks. MGIT tubes were incubated in the MGIT960 (BD Diagnostics) for 6 weeks. If any growth was detected, AFB was confirmed by performing Ziehl-Neelsen smears. NTM identification was performed by the GenoType NTM-DR ver1.0 line-probe (HAIN Lifescience, Germany) or laboratory-developed Sanger sequencing of 16S or *rpoB* as previously described (4).

When both methods were combined, 83 samples were positive and 89 organisms were detected (23.7% positivity, 89/376), with a positivity rate of 34.1% (75/220) from non-CF and 9.0% (14/156) from CF patients (Table 1). A total of 3 partially acid-fast bacteria (PAF), 26 rapidly growing (RGM), and 60 slowly growing (SGM) mycobacteria were detected (Table 2).

**TABLE 1:** Comparison of growth on NTM Elite agar, conventional acid-fast bacilli (AFB) culture, and when both methods were combined

		NTM Elite	Conventional AFB culture	Conventional AFB culture + NTM Elite
Sensitivity of organism detection	CF	69.2% (9/14)	92.3% (12/14)	100% (14/14)
	Non-CF	56.0% (42/75)	92.0% (69/75)	100% (75/75)
	All	57.3% (51/89)	91.0% (81/89)	100% (89/89)
Positivity rate (percentage of organisms detected/samples)	CF	5.8% (9/156)	7.7% (12/156)	9.0% (14/156)
	Non-CF	19.1% (42/220)	31.4% (69/220)	34.1% (75/220)
	All	13.6% (51/376)	21.5% (81/376)	23.7% (89/376)
Cultures with breakthrough bacterial contamination^*a*^	All	3.7% (14/376)	31.3% (118/376)	32.7% (123/376)
Average growth rate of SGM^*b*^	All	16.6 days	11.2 days	11.2 days
Average growth rate of RGM^*b*^	All	5.6 days	6.0 days	4.9 days

^*a*^ Note that this represents any contamination to show the rate of breakthrough bacteria; it does not represent a true contamination rate as defined by the CLSI M48 (5).

^*b*^ Out of 43 cultures that showed growth by both methods.

**TABLE 2:** Organisms recovered from conventional culture and NTM Elite

Organism	Type of organism^*a*^	No. grown from conventional culture	No. grown from NTM Elite
*Mycobacterium intracellulare*	SGM	23	14
*Mycobacterium avium*	SGM	21	7
*Mycobacterium abscessus* subsp. *abscessus*	RGM	12	12
*Mycobacterium abscessus* subsp. *massiliense*	RGM	5	4
*Mycobacterium intracellulare* subsp. *chimaera*	SGM	5	3
*Mycobacterium simiae*	SGM	4	3
*Mycobacterium fortuitum*	RGM	2	1
*Mycobacterium stomatepiae*	SGM	1	1
*Mycobacterium gordonae*	SGM	2	
*Nocardia nova*	PAF	2	
*Gordonia terrae*	PAF	1	
*Mycobacterium alvei*	RGM	1	
*Mycobacterium timonense*	SGM	1	
*Nocardia cyriacigeorgica*	PAF	1	
*Mycobacterium chelonae*	RGM		2
*Mycobacterium llatzerense*	RGM		2
*Mycobacterium marseillense*	SGM		1
*Mycobacterium paraterrae*	SGM		1
Total	All	81	51

^*a*^ SGM: slowly growing mycobacteria; RGM: rapidly growing mycobacteria; PAF: partially acid fast organism.

The sensitivity of conventional culture was 91.0% (81/89) but varied by media type: 77.5% (69/89) for MGIT broth, 76.4% (68/89) for 7H11/7H11 biplates, and 29.2% (26/89) for LJ. The sensitivity of NTM Elite alone was 57.3% (51/89) and was greatest for RGM (80.8%, 21/26) rather than SGM (50%, 30/60) or PAF (0%, 0/3).

Here, NTM Elite had higher sensitivity than LJ and, as previously seen in a recent European study, detected unusual SGM like *Mycobacterium llatzerense* that did not grow by conventional culture (6). However, previous publications showing higher positivity from NTM Elite (6–8) suggest that on-site evaluations should be performed to account for possible differences in patient populations, organisms, and laboratory practices (1, 9). For instance, different geographic regions have different proportions of organisms and susceptibility patterns (4, 10). NTM Elite also had low rates of breakthrough contamination (3.7% versus 31.3% for other solid media, though mycobacterial growth could still be evaluated in all cases due to the redundancy of media types). It is not designed to capture *M. tuberculosis*, and therefore growth may be suitable for workup under biosafety level-2 conditions. These factors suggest that NTM Elite may be a useful adjunctive or replacement media in laboratories with higher rates of RGM, or to capture unusual mycobacteria. However, the cost, changes to workflow, and additional labor should be considered.
